# Medication and the Risk of Falls: An Analysis of Adverse Drug Reactions Reported to the Portuguese Pharmacovigilance System

**DOI:** 10.3390/jcm12237268

**Published:** 2023-11-23

**Authors:** Daniela Rodrigues, Samuel Silvestre, Cristina Monteiro, Ana Paula Duarte

**Affiliations:** 1 Faculty of Health Science, University of Beira Interior, 6201-001 Covilhã, Portugal; daniela.n.rodrigues@ubi.pt; 2 UFBI-Pharmacovigilance Unit of Beira Interior, University of Beira Interior, 6201-001 Covilhã, Portugal; sms@ubi.pt (S.S.); apcd@ubi.pt (A.P.D.); 3 CICS-UBI-Health Sciences Research Centre, University of Beira Interior, 6201-001 Covilhã, Portugal

**Keywords:** adverse drug reactions, falls, Portuguese pharmacovigilance system, safety

## Abstract

Falls are not always considered direct adverse drug reactions (ADRs). However, due to their mechanism of action, certain drugs increase the risk of falls. This retrospective study aimed to evaluate the association between drugs and the risk of falls. An analysis of ADR reports submitted to a national pharmacovigilance database from 1992 to 2021 was performed using terms from the MedDRA dictionary. This included the word “fall” and terms related to conditions potentially predisposing patients to falls. The analysis involved examining the sex and age distribution of the population. Reports were assessed for seriousness, the class of the suspected drug, and the characterisation of fall events when they occurred. Over this period, 2217 cases were reported, with the majority occurring among females (60.71%) and the age group of 18–64 years old (38.43%). Most reports were classified as serious across all age groups, and immunomodulators (16.78%) were the most frequently reported pharmacotherapeutic class of suspected drugs. Falls were reported as ADRs in 343 cases, with fractures being the most commonly reported injuries (24.45%). In conclusion, falls can pose a significant health problem. Therefore, continuously monitoring drugs is crucial to minimise fall-associated risk factors.

## 1. Introduction

Adverse drug reactions (ADRs) can significantly impact health and must be reported for continuous vigilance by the authorities to ensure drug safety [1]. However, most ADRs, such as falls, are underreported. In fact, falls are not always seen as direct drug side effects [1]. According to the American [2] and British Geriatrics Societies [3], falls are defined as unexpected floor contacts without consciousness loss and can have a significant impact on the national health system [4].

A fall is the result of a complex interaction of risk factors, with drug use being one of the most modifiable. Due to their mechanism of action, some drugs may increase the risk of falls independently of other factors [5]. Some authors defined this group of drugs as fall-risk-increasing drugs (FRIDs), and they include medicines that affect the central nervous system (CNS), namely psychotropics, antiepileptics, antiparkinsonics, and antispasmodics. Additionally, some drugs used for heart-related conditions, such as diuretics, are also classified as FRIDs [4,5,6].

Psychotropics and tricyclic antidepressants can cause sedation, hypotension, and confusion, which elevate the risk of falls [4,6]. The relationship between antiepileptic drugs and falls is unclear, thus making it difficult to relate this situation with drug effects or seizures. However, despite the limited evidence connecting antiepileptics to falls, they are categorised as FRIDs [4]. In Parkinson’s disease, instability, rigidity, and bradykinesia heighten fall risk. In addition, the therapy for Parkinson’s disease can also exacerbate falls. In fact, antiparkinsonian drugs increase dopamine levels, which cause hypotension via dopaminergic receptors [6]. Moreover, antispasmodics notably raise the risk of falls among the elderly by inducing CNS depression, causing dizziness, hypotension, blurred vision, and confusion [5]. Furthermore, cardiovascular drugs, including antihypertensive agents, can also prompt the risk of falls by inducing gait disorder, disequilibrium, dizziness, and orthostatic hypotension [4].

Hence, the careful follow-up of patients taking FRIDs, particularly those causing dizziness, sedation, and hypotension, is essential to prevent falls [6]. In this vein, pharmacovigilance can be a determining factor in fall prevention via the communication of risks associated with these drugs and the establishment of measures to prevent this ADR. In fact, the use of FRIDs should be monitored and even limited, particularly in vulnerable groups [7]. As falls are not always considered a direct drug effect, it is essential to continue monitoring this ADR by gathering real-world evidence on the occurrence of serious reports and evaluating their impact. This study aims to analyse 29 years of Portuguese pharmacovigilance system (PPS) data to characterise ADRs linked to falls in the general population, considering that pharmacovigilance makes obtaining data in a real context possible. Furthermore, the secondary objective aims to apprise healthcare professionals and patients of potential fall occurrences using terminology distinct from the term “fall”.

## 2. Materials and Methods

The study’s data emanate from reports acquired by the PPS, accessible within INFARMED, I.P.’s centralised database, known as the “Portal RAM”. This observational and descriptive study encompassed reports spanning from 1992 to 2021, encompassing all instances of ADR with the potential to precipitate a fall.

Initially, 2884 reports were identified within the PPS database. After removing duplicates and reports featuring anaphylactic reactions omitted due to their non-correlation with the chronic use of a drug, as falls resulting from such reactions are independent of the pharmacological effects and inherently unpredictable, 2217 reports were selected for comprehensive analysis.

Data were evaluated through descriptive statistical methods, including relative frequency, absolute frequency, and percentages, facilitated by Microsoft^®^ Office^®^ Excel^®^ 365 software (Microsoft Corporation, Washington, DC, USA).

### 2.1. Population

The study population comprised cases of suspected falls associated with ADR that were spontaneously reported to PPS.

The identification of these reports relied on the Medical Dictionary for Regulatory Activities (MedDRA). Specifically, it targeted the following high-level terms (HLTs): “Fall”, “Falling”, “Falling down”, “Disequilibrium”, “Gait abnormal”, “Gait abnormal NOS”, “Gait disorder”, “Gait disturbance”, “Gait instability”, “Vertigo”, “Vertigo (excl. dizziness)”, “Vertigo aggravated”, “Visual acuity decreased”, “Visual acuity lost”, “Visual acuity reduced”, “Visual disturbance”, “Visual disturbances”, “Visual disturbance NOS”, “Visual impairment”, “Balance disorder”, “Vestibular abnormalities”, “Vestibular disorder”, “Vestibular vertigo”, “Disequilibrium syndrome”, “Hypotension”, “Hypotension aggravated”, “Hypotension orthostatic”, “Hypotension postural”, “Orthostatic hypotension”, “Postural hypotension”, “Hypotension postural aggravated”, “Hypotension orthostatic asymptomatic”, and “Hypotension orthostatic symptomatic”.

The search terms used in the portal were derived from current studies and from scientific evidence identifying drug-related adverse reactions associated with an elevated risk of falls. These terms are related reactions linked to ADRs, acknowledging that while some may not directly cause falls, they can create conditions that lead to a fall [8,9,10].

### 2.2. Variables

#### 2.2.1. Demographic Characterisation of the Population

Demographic analysis of patients encompassed distinct age groups: 2 months to 2 years old; 3–11 years old; 12–17 years old; 18–64 years old; >65 years. Sex was classified as male, female, and unknown.

#### 2.2.2. Characterisation of the Report Rate and Report Source

The data were stratified based on the reporting rate spanning from 1992 to 2021 and the source of ADR reports: physicians, pharmacists, nurses, other healthcare professionals, consumers, or other non-healthcare professionals or marketing authorisation holders.

#### 2.2.3. Characterisation of ADR Reports

The data were categorised according to the patient’s clinical status (cure, cure with sequelae, recovery, persistence without recovery, death, and unknown) and based on the seriousness (serious and non-serious). Serious ADRs were further stratified at clinically important, hospitalisation, incapacity, congenital anomaly, risk to life, and death.

Correlations were established among the data, considering age group and sex in relation to seriousness. Additionally, serious ADR reports were correlated with the route of administration (oral use, parenteral use, inhalation use, topical use, ocular use, multiple ways, or unknown).

The drugs reported were evaluated according to pharmacotherapeutic classes [11]. An analysis of other terms present regarding ADRs that may contribute to a fall was also performed in the study.

#### 2.2.4. Characterisation of Fall ADRs

An analysis was made based on the reports that included only the terms “Fall”, “Falling”, and “Falling down”. These data were stratified according to seriousness (serious or non-serious). Serious ADRs associated with these terms were characterised by the type of injury (fractures, superficial injuries, head injury, dislocations, sprains, contusions, stretching of joints and ligaments, open injuries, no injury, death, or undescribed) and the body region affected (head, hip and thigh, lower limb, trunk, upper limb, multiple, and unspecified body region). Drugs that caused a fall were evaluated according to pharmacotherapeutic classes [11].

## 3. Results

### 3.1. Reporting Rate

**Figure 1:**
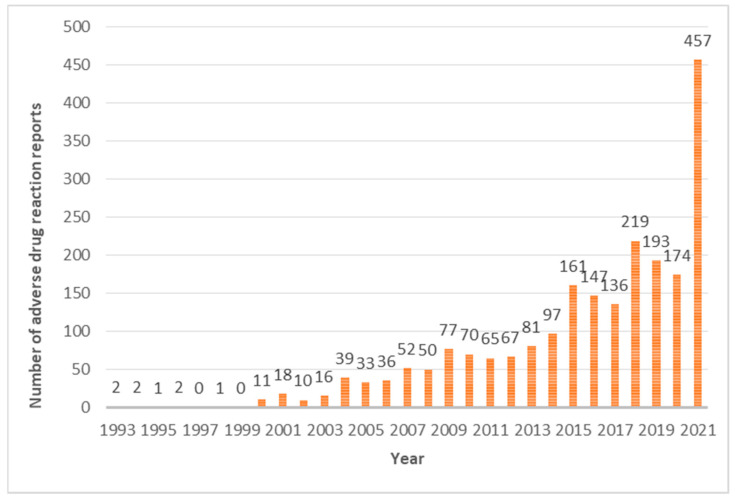
Number of reports received each year.

Reporting generally increased over the years, especially during the last year analysed, as shown in Figure 1.

### 3.2. Demographic Analysis

**Figure 2:**
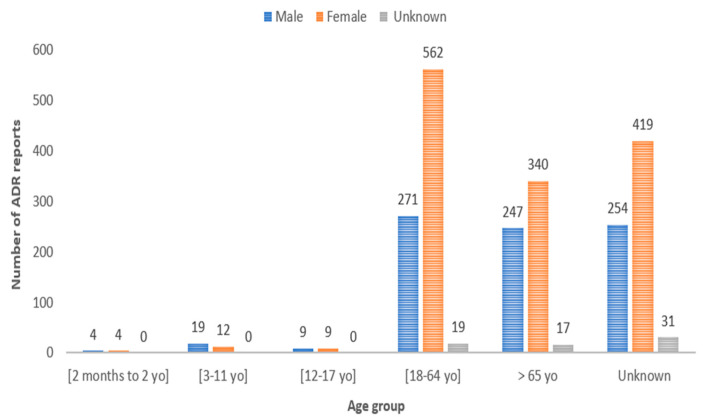
Characterisation of the population in terms of sex and age group.

Analysis of patient demographics, including age range and sex, is presented in Figure 2. Most participants (*n* = 852; 38.43%) fell within the age range of 18 to 64 years old. Females accounted for the highest number of reports (*n* = 1346; 60.71%).

### 3.3. Source of Reports and Adverse Drug Reaction Outcomes

Marketing authorisation holders submitted 1118 out of 2217 (50.43%) reports. The remaining 1099 out of 2217 (49.57%) originated from physicians, pharmacists, nurses, other healthcare professionals, consumers, or other non-healthcare professionals. Among these, physicians (*n* = 435; 39.58%) were the primary contributors to ADR reports, followed by pharmacists (*n* = 365; 33.21%).

The outcomes of the patients’ clinical status were reported in 1447 out of 2217 reports (65.27%), while in 770 out of 2217 cases (34.73%), the outcomes remained unknown. Notably, in the suspected ADR progressed to cure in 45.15% (*n* = 1001) of the reports.

### 3.4. Adverse Drug Reaction Seriousness and Causality

Most ADRs reported were classified as serious, totalling 74.02% (*n* = 1641) of all analysed reports. Additionally, 44.42% (*n* = 729) of the reported ADRs were classified as clinically important.

#### 3.4.1. Relation between Age Group, Gender, and Seriousness

**Figure 3:**
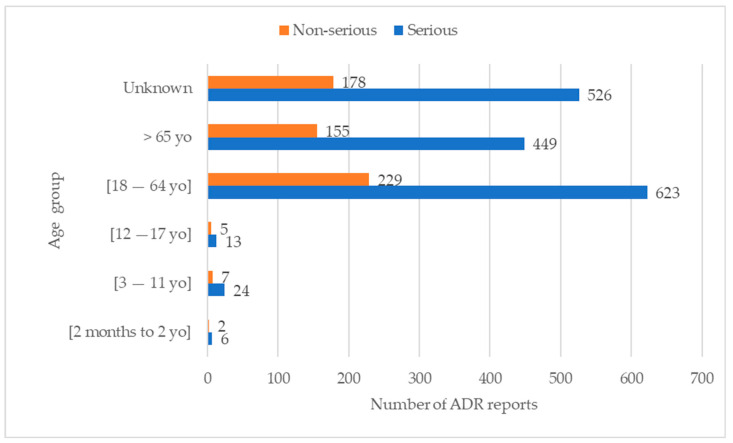
Relation between age and seriousness of adverse drug reaction reports.

Females account for the majority of these reports (*n* = 971; 43.80%). In addition, the highest incidence of serious ADR reports, comprising 623 cases (28.10%), was observed within the age range of 18 to 64 years old (Figure 3). Furthermore, most ADR reports were categorised as serious across all age groups.

#### 3.4.2. Routes of Administration Mostly Associated with the Occurrence of Serious Adverse Drug Reactions

**Figure 4:**
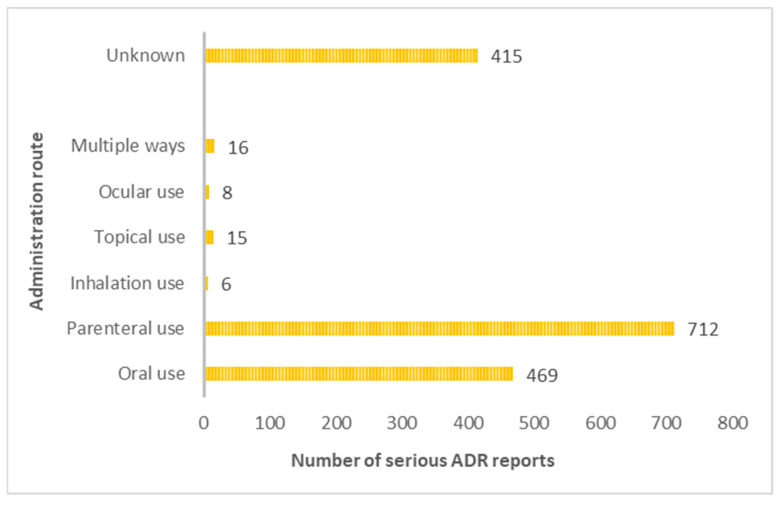
Relation between the route of administration of the suspected drug and the occurrence of a serious reaction.

While the route of administration was not specified in a significant percentage of ADR reports (*n* = 415; 25.29%), among the serious reports with a known route of administration, the parenteral route emerged as the most prevalent (*n* = 712; 43.39%), Figure 4.

### 3.5. Pharmacotherapeutic Classes and Suspected Drugs Associated with the Risk of Falls

Specific medications were identified more frequently as potential triggers for ADR-related falls. Table 1 presents the most commonly implicated drugs. A significant part of the analysed reports (12.00%, *n* = 266) pointed to the involvement of multiple medications as potential contributors to ADR, particularly associations involving psychoactive drugs. The remaining classes, with less than 1% of incidences, were collectively categorised as “Others”, constituting 15.47% (*n* = 307).

### 3.6. Reports Mentioning the Occurrence of a Fall

Among the reports, 343 out of 2217 highlighted falls as ADRs, encompassing terms including “Fall”, “Falling”, and “Falling down”. The cases where a fall occurred were analysed, and the associated drugs are detailed in Table 2. Notably, a significant part of the analysed reports (12.54%, *n* = 43) implicated multiple drugs as potential contributors to falls. The remaining classes, each with incidences of less than 1%, were grouped as “Others”, constituting 9.62% (*n* = 33) (Table 2).

**Figure 5:**
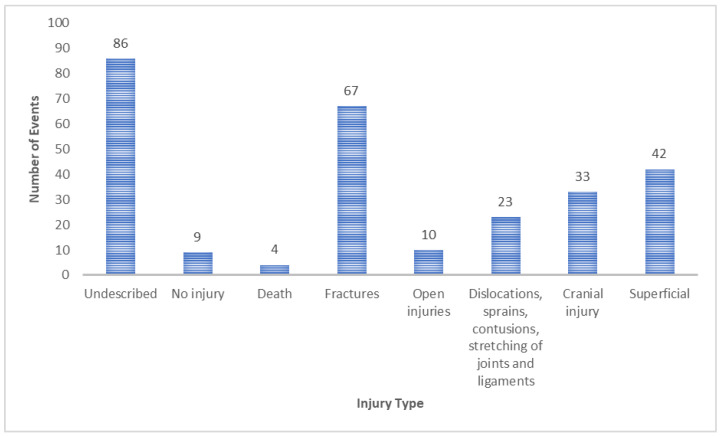
Type of injuries caused by falls of serious reports.

In the context of these 343 out of 2217 reports, the majority comprised serious ADRs (*n* = 243; 70.85%). Our analysis focused on scrutinising the serious reports to ascertain both the type and location of injuries. Multiple injuries were delineated in 274 clinical events, as illustrated in Figure 5. The type of injury was documented in 188 instances, while the remaining cases (*n* = 86) lacked any elucidation regarding the type of injury or the affected region after a fall.

Table 3 shows the regions affected post-fall categorised by the type of injury.

Superficial injuries include specific symptoms such as pain, bruising, oedema, and minor wounds (scratches). Head injuries include intracranial, subdural, and subarachnoid haemorrhages, as well as head trauma and haematomas.

### 3.7. Other Adverse Drug Reactions Associated with a Fall

We examined reports to identify potential factors contributing to falls, even when not explicitly stated. The analysis encompassed terms suggesting conditions that increase the risk of falls. A single report could involve multiple reactions and terms. Hypotension was relatively common (*n* = 977; 28.57%) among the factors associated with falls, as shown in Table 4.

## 4. Discussion

Our study evidences an increasing trend in notification rates, particularly evident in 2021, in accordance with INFARMED data. This increase is probably due to the enhanced accessibility of “Portal RAM” since 2017. The creation of “Portal RAM” aimed to facilitate the process of reporting side effects by digitising all relevant information, thus facilitating the input of ADRs in a straightforward, accessible, and prompt manner, eliminating the need for third-party intermediation. The implementation of the portal has led to an increase in reports [12,13].

The rise observed in 2021 can be attributed to the impact of the COVID-19 vaccine, which has highlighted both public and professional awareness, consequently leading to a substantially increased number of ADR reports [12,13]. In fact, vaccine-related reports ranked as the second most frequent (refer to Table 1 and Table 2), thus substantiating this observation. Concerning patient demographics, the 18–64 age group predominated in the reports, aligning with the population distribution in Portugal [4,14,15]. Regarding gender distribution, the study included more women, which is also in line with the Portuguese population data [16]. In addition, increased healthcare utilisation by women and sex-specific pharmacokinetic/pharmacodynamic differences heighten susceptibility to ADRs [17,18]. In this context, it becomes evident that women exhibit higher body fat proportions than men. This leads to an augmented retention of slowly metabolised fat-soluble substances in the female body, thus contributing to an elevated body burden of these substances. Consequently, this heightened body burden increases the potential for ADRs among women [17]. Most reports originated from marketing authorisation holders (50.43%) [19]. Furthermore, physicians led the submission of reports, closely followed by pharmacists, aligning with findings from previous studies [20,21]. This trend may stem from physicians’ direct interactions with patients and the pharmacists’ role as first contact with healthcare users [21]. Across all age groups, most ADRs were deemed serious, possibly reflecting the heightened vigilance of health professionals in reporting such reactions, although a significant percentage resolved favourably [22]. Moreover, serious ADRs were more prevalent in women, which is consistent with the female predominance in the study. Notably, parenteral drug administration predominantly triggered serious ADRs, a phenomenon potentially explicable by considering the drug classes associated with fall-related ADRs: immunomodulators and vaccines [23]. Adverse effects, such as vision disturbances and general body reactions, may elucidate their prevalence in fall-related reports [23]. For example, an analysis of the summary of product characteristics (SmPC) for immunomodulators like infliximab, adalimumab, and rituximab reveals ADRs, such as dizziness and hypotension. However, none of these SmPCs mentioned falls as potential side effects [24,25,26]. In fact, the absence of explicit mention of “falls” in some SmPCs may contribute to health professionals and patients underestimating the risk, potentially resulting in a lack of necessary precautions. Consequently, issuing warnings about the risk of falls related to these medications could be beneficial to avoid this ADR [27]. In addition, it is crucial to consider that some of the most frequently mentioned drugs are used for diseases that, in turn, may lead to falls. This consideration is imperative to mitigate these medications’ heightened risk of falls. Considering vaccines, particularly COVID-19 vaccines, they have been systematically the object of significant reporting within the PPS [21]. In fact, COVID-19 vaccines, subject to additional monitoring by the European Medicines Agency, have attracted considerable attention due to safety concerns [28]. Similar to immunomodulators, vaccines can affect equilibrium, thereby increasing the risk of falls [29,30,31]. Moreover, the hypotension, syncope, and visual disturbances reported for both vaccines and immunomodulators may be mainly related to intramuscular administration/pain rather than constitute a “drug-specific” side effect. Considering FRIDs, it is important to mention that the SmPCs of some psychotropics (e.g., quetiapine, diazepam) and antiepileptics (e.g., pregabalin) explicitly indicate falls, a feature possibly attributed to the more vigilant monitoring of FRID classes [32,33,34]. However, it is essential to acknowledge that the classes included in the FRIDs may not be the most frequently reported classes for falls, thereby underscoring the issue of underreporting associated with many fall episodes [15]. In addition, considering that patients often use multiple drugs simultaneously to address various health conditions, the interaction between these drugs can complicate attributing a fall to a specific drug class [35]. In Portugal, more than 117,000 notifications were reported until 2021, and the present study revealed only 343 falls [13]. Similarly, the Spanish pharmacovigilance system documented 232 fall-related ADRs among 347,000 notifications received in 2019 [15,36,37]. This pronounced underreporting suggests that the results may not comprehensively reflect the actual situation in clinical practice. Underreporting and its associated biases pose a significant problem in realising the epidemiological potential of incident reporting within healthcare [27]. In this study, the reports of serious falls align with previous findings, predominantly involving fractures followed by superficial injuries [36]. Hence, vigilance is essential, namely that carried out by physicians, especially for individuals at a higher risk of falling [38,39,40]. In Canada, an annual occurrence of falls affects 20% of individuals over the age of 65, resulting in substantial hospitalisations among seniors aged 80 and older [41]. In addition to falls as an ADR, other ADRs can also lead to a fall, and particular attention should be directed towards reports containing the additional terms identified in Table 4. In such cases, patients should be regularly questioned about the occurrence of falls attributed to vertigo, dizziness, or any alteration in the state of consciousness following drug administration. This study has some limitations, such as the underreporting of ADRs, such as falls, which are not considered adverse drug effects. However, underreporting is common in pharmacovigilance [42], with non-serious cases being particularly prone to underreporting. In addition to underreporting, another limitation is the quality of the reports. Many lacked essential details (e.g., concurrent medications, patient medical history, and environmental conditions) essential for a nuanced analysis of the causality of falls [41]. Genetic, inter-individual, and external factors can also influence the observed results [43]. Genetic variations, for instance, may significantly impact ADR susceptibility, while variables such as weight and gender can influence drug response. External factors such as health conditions, environment, and lifestyle also contribute to result variability [43]. Additionally, the research is based on information gathered from a national pharmacovigilance database, introducing the potential for selection bias. This raises concerns about an incomplete representation of the entire population, especially among individuals who did not seek medical assistance for falls attributed to drug reactions, thereby affecting our ability to accurately ascertain the actual frequency and attributes of falls linked to medication.

## 5. Conclusions

Falls pose a significant health problem. Most ADR reports associated with falls were classified as serious, with immunomodulators being the most frequently implicated pharmacotherapeutic class. ADR manifestations, such as dizziness, vertigo, vision disturbances, hypotension, bradycardia, and altered states of consciousness, may mask the recognition of a falling episode. Therefore, implementing preventive programs is crucial to reduce the incidence of fall-related injuries as a consequence of ADRs. The importance of pharmacovigilance in understanding the safety of drugs, particularly regarding the risk of falls, emphasises the need for vigilant reporting by all healthcare professionals to ensure accurate information.

## Figures and Tables

**Table 1:** Pharmacotherapeutic classes of drugs primarily associated with the risk of falls.

Pharmacotherapeutic Classification	Drugs	*n* (%)
Immunomodulators	Glatiramer acetate; Adalimumab; Alemtuzumab; Atezolizumab, Bevacizumab; Certolizumab pegol; Cetuximab; Cyclosporine; Daratumumab; Etanercept; Everolimus fingolimod; Dimethyl fumarate; Golimumab; Infliximab; Interferon beta-1a; Interferon beta-1b; Interferon gamma-1b; Ipilimumab; Lenalidomide; Natalizumab; Nivolumab; Obinutuzumab; Ocrelizumab; Peginterferon beta-1a; Pembrolizumab; Pertuzumab; Pirfenidone; Rituximab; Tacrolimus; Thalidomide; Teriflunomide; Tocilizumab; Trastuzumab; Ustecinumab	372 (16.78%)
Vaccines (single and conjugate)	Diphtheria and tetanus vaccine; Influenza vaccine; Pandemic influenza vaccine; Adsorbed pneumococcal polyacid conjugate vaccine; Hepatitis B vaccine; Human papillomavirus vaccine; Human papillomavirus vaccine (types 6, 11, 16, 18); Human papillomavirus vaccine (types 6, 11, 16, 18, 31, 33, 45, 52, 58); Tetanus vaccine; Meningococcal vaccine; Measles, mumps, rubella vaccine; mRNA vaccine against COVID-19 (with modified nucleoside); COVID-19 non-replicating vectored viral vaccine (chimpanzee adenovirus); COVID-19 non-replicating vectored viral vaccine (human adenovirus type 26); Live rotavirus vaccine	359 (16.19%)
Cytotoxics	Alectinib; Axicabtagene ciloleucel; Axitinib; Bleomycin; Bortezomib; Bosutinib; Carboplatin; Cisplatin; Capecitabine; Cladribine; Crizotinib; Dasatinib; Radium dichloride (223Ra); Docetaxel; Doxorubicin; Erlotinib; Exemestane; Etoposide; Glasdegib; Idelalisib; Irinotecan; Lenvatinib Methotrexate; Mitotane; Nilotinib; Nintedanib; Osimertinib; Oxaliplatin; Paclitaxel; Pazopanib; Pegaspargase; Regorafenib; Ribociclib; Ruxolitinib; Selumetinib; Sorafenib; Sunitinib; Verteporfin	125 (5.64%)
Psychoactive drugs	Agomelatine; Alprazolam; Amitriptyline; Aripiprazole; Bupropion; Chlorpromazine; Cloxazolam; Clozapine; Dexmedetomidine; Diazepam; Duloxetine; Escitalopram; Fluoxetine; Fluvoxamine; Haloperidol; Levomepromazine; Lithium; Loflazepat ethyl; Lorazepam; Melatonin; Mexazolam; Mirtazapine; Olanzapine; Paliperidone; Paroxetine; Quetiapine; Risperidone; Sertraline; Sulpiride; Temazepam; Trazodone; Valerian; Venlafaxine; Ziprasidone; Zuclopenthixol	105 (4.74%)
Antihypertensives	Altizide + Spironolactone; Amlodipine; Amlodipine + Valsartan; Amlodipine + Olmesartan medoxomilo; Amlodipine + Olmesartan medoxomilo + Hydrochlorothiazide; Bisoprolol; Bisoprolol + Perindopril; Candesartan + Hydrochlorothiazide; Captopril; Carvedilol; Cilazapril; Cilazapril + Hydrochlorothiazide; Chlorthalidone; Doxazosin; Enalapril + Hydrochlorothiazide; Eplerenone; Spironolactone; Furosemide; Hydrochlorothiazide + Triamterene; Indapamide; Indapamide + Amlodipine; Irbesartan + Hydrochlorothiazide; Lercanidipine; Lisinopril; Lisinopril + Hydrochlorothiazide; Losartan; Methyldopa; Nebivolol; Nifedipine; Olmesartan medoxomilo; Olmesartan medoxomilo + Hydrochlorothiazide; Perindopril; Perindopril + Amlodipine; Perindopril + Indapamide; Propranolol; Ramipril; Riociguat; Sacubitril + Valsartan; Telmisartan + Hydrochlorothiazide; Valsartan; Valsartan + Hydrochlorothiazide	104 (4.69%)
Antibacterial drugs	Amoxicillin; Amoxicillin + Clavulanic acid; Azithromycin; Benzylpenicillin benzathine + Benzylpenicillin potassium + Benzylpenicillin procaine; Cefatrizine; Cefazolin; Cefuroxime; Cefotaxime; Cefotetan; Cefoxitin; Cefradin; Ceftazidime; Ceftazidime + Avibactam; Ceftriaxone; Ciprofloxacin; Clarithromycin; Clindamycin; Flucloxacillin; Imipenem + Cilastatin; Isoniazid; Levofloxacin; Metronidazole; Minocycline; Moxifloxacin; Ofloxacin; Piperacillin + Tazobactam; Rifampicin; Bismuth potassium subcitrate + Metronidazole + Tetracycline; Sulfamethoxazole + Trimethoprim; Teicoplanin; Telithromycin; Tinidazole; Vancomycin	94 (4.24%)
Anticoagulants and Antithrombotics	Acenocoumarol; Acetylsalicylic acid; Alteplase; Apixaban Clopidogrel; Dabigatran etexilate; Enoxaparin sodium; Heparin sodium; Iloprost; Rivaroxaban; Tenecteplase; Ticagrelor; Ticlopidine; Warfarin	78 (3.52%)
Analgesics and antipyretics	Acetylsalicylic acid; Chlorphenamine + Paracetamol; Delta-9-tetrahydrocannabinol (THC PFV) + Cannabidiol (CBD PFV), Prep from plant drugs, ext Cannabis sativa; Flupirtine; Gabapentin; Metamizole magnesium; Metamizole sodium; Paracetamol; Pregabalin	54 (2.44%)
Non-steroidal anti-inflammatory drugs	Acemetacin; Cetorolac; Colecoxib; Diclofenac; Diclofenac + Misoprostol; Etodolac; Etoricoxib; Flurbiprofen; Ibuprofen; Indomethacin; Lumiracoxib; Naproxen; Naproxen + Esomeprazole; Nimesulide; Rofecoxib	43 (1.94%)
Antiarrhythmics	Amiodarone; Diltiazem; Propafenone; Sotalol; Verapamil	37 (1.67%)
Other central nervous system drugs	Disulfiram; Donepezil; Fampridine; Ginkgo biloba; Memantine; Rivastigmine; Varenicline; Vinpocetine	36 (1.62%)
Antivirals	Abacavir + Lamivudine; Acyclovir; Amphotericin B; Boceprevir; Dolutegravir + Abacavir + Lamivudine; Efavirenz; Efavirenz + Emtricitabine + Tenofovir; Elvitegravir + Cobicistat + Emtricitabine + Tenofovir; Emtricitabine + Tenofovir; Entecavir; Ledipasvir + Sofosbuvir; Oseltamivir; Raltegravir; Remdesivir; Ribavirin; Ritonavir; Sofosbuvir; Sofosbuvir + Velpatasvir; Tenofovir; Valacyclovir; Voriconazole	34 (1.53%)
Hormones and antihormones	Anastrozole; Apalutamide; Bicalutamide; Enzalutamide; Goserreline; Letrozole; Leuprorreline; Megestrol; Tamoxifen; Triptorreline	34 (1.53%)
Antianemics	Ferric carboxymaltose; Cobamamide; Iron–dextran complex; Saccharose ferric oxide; Ferrous sulphate + Folic acid	32 (1.44%)
Insulin, antidiabetics, and glucagon	Dapagliflozin; Diazóxido; Dulaglutido; Empagliflozin; Ertugliflozin; Exenatido; Glibenclamida; Glibenclamida + Metformin; Gliclazida; Glimepirida + Pioglitazona; Insulin degludec; Insulin humana; Insulin isofânica; Linagliptin; Metformin; Metformin + Pioglitazona; Metformin + Sitagliptin	31 (1.40%)
Radiologic contrast media	Iobitridol; Iodixanol; Io-hexol; Iomeprol; Iopromide; Ioversol	30 (1.35%)
Antiepileptics and anticonvulsants	Eslicarbazepine acetate; Valproic acid; Carbamazepine; Phenytoin; Gabapentin; Lacosamide; Levetiracetam; Perampanel; Pregabalin; Primidone; Topiramate; Valproate semisodium; Zonisamide	28 (1.26%)
Drugs that act on the bone and calcium metabolism	Alendronic acid + Colecalciferol; Ibandronic acid; Zoledronic acid; Ibandronic acid + Colecalciferol; Colecalciferol; Denosumab; Raloxifene; Strontium ranelate; Teriparatide	25 (1.13%)
Other drugs used in genitourinary disorders	Alfuzosin; Throspium chloride; Doxazosin; Dutasteride + Tansulosin; Finasteride; Oxybutynin; Silodosin; Tadalafil; Tansulosin; Terazosin; Vardenafil	23 (1.04%)
Others		307 (15.47%)

**Table 2:** Pharmacotherapeutic classes of drugs associated with reports of falls.

Pharmacotherapeutic Classification	Drugs	*n* (%)
Immunomodulators	Adalimumab; Bevacizumab; Fingolimod; Dimethyl fumarate; Golimumab; Interferon beta-1a; Interferon beta-1b; Interferon gamma-1b; Ipilimumab; Natalizumab; Peginterferon beta-1a; Pirfenidone; Rituximab; Tocilizumab	99 (28.86%)
Vaccines (single and conjugate)	Adsorbed pneumococcal polyoside conjugate vaccine; Diphtheria and tetanus vaccine; COVID-19 mRNA vaccine (with modified nucleoside); COVID-19 non-replicating viral vector vaccine (chimpanzee adenovirus); COVID-19 non-replicating viral vector vaccine (human adenovirus type 26); Pneumococcal conjugate vaccine	47 (13.70%)
Anticoagulants and Antithrombotics	Acenocoumarol; Acetylsalicylic acid; Apixaban; Dabigatran etexilate; Enoxaparin sodium; Rivaroxaban; Warfarin	34 (9.91%)
Psychoactive drugs	Agomelatine; Chlorpromazine; Duloxetine; Loflazepato de etilo; Lorazepam; Mirtazapine; Olanzapine; Paliperidona; Quetiapine; Risperidona; Sertraline; Trazodona; Zuclopentixol	24 (7.00%)
Other central nervous system drugs	Donepezil; Fampridine; Rivastigmine	12 (3.50%)
Insulin, antidiabetics, and glucagon	Ertugliflozin; Exenatide; Glibenclamide; Gliclazide; Insulin degludec; Insulin isophane; Linagliptin; Metformin; Sitagliptin	9 (2.62%)
Antiepileptics and anticonvulsants	Valproic acid; Carbamazepine; Perampanel; Pregabalin; Primidone; Zonisamide	8 (2.33%)
Antihypertensives	Hydrochlorothiazide + Triamterene; Olmesartan medoxomilo; Perindopril + Indapamide; Sacubitril + Valsartan	7 (2.04%)
Drugs that act on bone and calcium metabolism	Alendronic Acid + Cholecalciferol; Denosumab; Raloxifene; Teriparatide	7 (2.04%)
Antiparkinsonians	Levodopa + Carbidopa; Opicapona	6 (1.75%)
Cytotoxics	Cladribine; Idelalisib; Lenvatinib; Nintedanib; Ribociclib; Ruxolitinib	6 (1.75%)
Antivirals	Entecavir; Ledipasvir + Sofosbuvir	4 (1.17%)
Hormones and antihormones	Enzalutamide; Goserrelin; Letrozole; Triptorrelin	4 (1.17%)
Others		33 (9.62%)

**Table 3:** Types of injuries and corresponding affected body regions.

Type of Injury	Affected Area	*n* (%)
Fractures	Head	4 (1.46%)
	Hip and thigh	8 (2.92%)
	Lower limb	31 (11.31%)
	Trunk	12 (4.38%)
	Upper limb	10 (3.65%)
	Multiple and unspecified body region	2 (0.73%)
Superficial	Head	14 (5.11%)
	Hip and thigh	1 (0.36%)
	Lower limb	7 (2.55%)
	Trunk	6 (2.19%)
	Upper limb	7 (2.55%)
	Multiple and unspecified body region	7 (2.55%)
Head injury	Head	33 (12.04%)
Dislocations, sprains, contusions, stretching of joints and ligaments	Head	2 (0.73%)
	Hip and thigh	1 (0.36%)
	Lower limb	7 (2.55%)
	Trunk	1 (0.36%)
	Upper limb	5 (1.82%)
	Multiple and unspecified body region	7 (2.55%)
Open injuries	Head	6 (2.19%)
	Hip and thigh	0
	Lower limb	2 (0.73%)
	Trunk	0
	Upper limb	1 (0.36%)
	Multiple and unspecified body region	1 (0.36%)
No injury		9 (3.28%)
Death		4 (1.46%)
Undescribed		86 (31.39%)

**Table 4:** Other ADRs that can cause a fall.

Other ADRs ^a^ Associated with Falls	*n* (%)
Hypotension	977 (28.57%)
Visual disturbances ^b^	532 (15.56%)
Gait disorders ^c^	529 (15.47%)
Dizziness	377 (11.02%)
Vertigo	358 (10.47%)
Altered state of consciousness ^d^	150 (5.70%)
Syncope	188 (5.50%)
Bradycardia	118 (3.45%)
Sleepiness	75 (2.19%)

^a^ ADR: Adverse Drug Reaction; ^b^ Visual disturbance included ADRs such as reduced Visual acuity, Visual impairment, Eye disorder, and blurred Vision. ^c^ Gait disorders included terms such as Gait disturbance, Balance disorder, Coordination abnormal, decreased Mobility, and Movement disorder; ^d^ Altered state of consciousness included: Altered state of consciousness, Loss of consciousness, Confusional state, Depressed level of consciousness, and Disorientation.

## Data Availability

The raw data used in this research are available from the authors, depending on INFARMED’s authorisation.
